# Research on a Gas Concentration Prediction Algorithm Based on Stacking

**DOI:** 10.3390/s21051597

**Published:** 2021-02-25

**Authors:** Yonghui Xu, Ruotong Meng, Xi Zhao

**Affiliations:** Institute of Automatic Testing and Control, Harbin Institute of Technology, Harbin 150080, China; rtmhit@163.com (R.M.); richard214@163.com (X.Z.)

**Keywords:** ensemble learning, model fusion, regression algorithm, automatic grid search algorithm

## Abstract

Machine learning algorithms play an important role in the detection of toxic, flammable and explosive gases, and they are extremely important for the study of mixed gas classification and concentration prediction methods. To solve the problem of low prediction accuracy of gas concentration regression prediction algorithms, a gas concentration prediction algorithm based on a stacking model is proposed in the current research. In this paper, the stochastic forest, extreme random regression tree and gradient boosting decision tree (GBDT) regression algorithms are selected as the base learning devices and use the stacking algorithm to take the output of each base learning device as input to train a new model to produce a final output. Through the stacking model, the grid search algorithm is studied to automatically optimize the parameters so that the performance of the entire system can reach the optimal parameters. Through experimental simulation, the gas concentration prediction algorithm based on stacking model has better prediction effect than other integrated frame algorithms and the accuracy of mixed gas concentration prediction is improved.

## 1. Introduction

With the rapid development of artificial intelligence, sensor applications and machine learning algorithms promote the continuous development of machine olfaction [1]. Machine olfaction is a new type of bionic detection technology, which is utilized to simulate the working mechanism of biological olfactory systems [2]. Machine olfaction is widely used in pollution control [3,4], medical technology [5,6], oil exploration [7] and other fields. In the study of machine olfaction, researchers have achieved good results, and an important application area is the detection and analysis of dangerous gases such as flammable and explosive [8,9]. That is because the gas leakage problems will seriously endanger the safety of human life all over the world [10]. If these gases can be detected and classified accurately in time, and the leakage situation and trend can be calculated, the harm to humans can be minimized and the occurrence of dangerous accidents can be avoided to a great extent [11]. Therefore, machine olfaction is of great significance for the detection of toxic, flammable and explosive gases. At present, in the process of using machine olfactory technology to detect flammable, explosive and toxic gases, machine learning algorithms play a major role. A good algorithm is needed as the support to effectively identify or predict the gas information collected by the machine olfactory system. Accuracy and time efficiency of machine learning algorithms affect the performance of the entire system, therefore, it is extremely important to study the concentration prediction method of mixed gas.

There are two main problems in the research field of mixed gas concentration prediction. One is to construct the most effective system to collect gas information, and the other is to build the most suitable algorithm model for concentration regression prediction of the data set [12,13,14]. For the concentration regression prediction that we mentioned, it is a regression problem in the algorithm. Researching and designing the most suitable prediction algorithm for gas concentration represents a current research endeavor. At present, most of the main research directions are aimed at the field of classification of mixed gases, and they ignore the prediction of concentration [15]. There are two main difficulties in gas concentration prediction; one is in the process of collecting gas information—because the design of the collection system is more complex and the experimentation is more complicated, the experimental process consumes considerable time. Moreover, the gas configuration process is rather cumbersome, it is difficult to continuously conduct the gas concentration value experimentation. Therefore, discontinuous concentration data information will appear. Another difficulty lies in the processing stage of the algorithm—because the prediction of discrete data is already quite complicated, it is more difficult to produce a good concentration regression prediction of the data [16]. Thus, there are few studies on the concentration prediction of mixed gases currently. In relatively few studies, the electronic nose system based on a wireless sensing network, built by Zhao et al., quantitatively analyzes the gas composition information through the fuzzy neural network model based on RBF, and realizes the quantitative detection of mixed gas [17]. Devit et al. used an artificial neural network algorithm to design an intelligent electronic nose system to overcome interference problems through a large amount of training, with local recognition and quantification capabilities. The sensor fusion algorithm can be used to reconstruct the three-dimensional concentration of chemicals in the data link [18]. Reference [19] compares the quantitative detection performance of gas-based MLP with single multiple inputs multiple outputs and multiple multiple inputs single output, which improves the concentration detection accuracy for many kinds of single gases. Sikora M. et al. proposed a hybrid adaptive system for gas concentration prediction. The system has the ability to automatically disconnect power in the event of explosion hazard identification. The first part of the prediction system uses linear and nonlinear prediction methods and it uses a solution of metalearning to negotiate the system final forecast between linear and nonlinear methods. The second part of the system uses a knowledge base of typical situations that lead to explosion or fire risk increase to monitor the time series of methane and other gas concentrations from the perspective of the similarity of the current time series [20]. Reference [21] addresses power load, coal load level, emission gas concentration, total pressure and temperature dynamic changes in power plants. The main purpose is to control the coal feed, water supply and bed height through a series of gas concentration predictions. It can quickly respond and control the dynamically changing exhaust gas.

In this paper, based on the integrated learning method, the mixed gas concentration prediction algorithm is studied. The integrated learning algorithm can greatly improve the detection and analysis performance of the machine olfactory system by combining a variety of learners and using various rules to merge them together [22]. Integrated learning is a machine learning method in which numerous functions are trained and combined, usually performing classification or regression tasks. Overall, the integration process is mainly composed of integration generation and integration [23]. Simply speaking, integrated learning is the process of integrating multiple algorithms and ultimately forming a complete algorithm through specific rules. Integrated learning primarily involves base learning devices. The general framework of integrated learning is mainly composed of base learning devices. In the data set, the base learning devices are assigned training data sets through different rules, allowing each base learning device to perform algorithm operations, and then performing algorithm fusion integration according to the fusion integration rules; that is, forming an integrated model to get a strong learning machine [24]. In the integrated generation, there are different sampling rules, including uniform sampling, systematic sampling and weighted sampling like boosting [25]. In the fusion rules, there is a method for decision trees to vote like the random forest algorithm, and there are also serial fusion rules like the stacking model. Different integration rules will create different integrated learning methods [26]. The strong learning machine obtained through integrated learning tends to have better effects and stronger capabilities. Finally, the integrated algorithm model is tested according to the test sample data set to obtain the final prediction effect.

In this paper, a gas concentration regression prediction algorithm based on stacking model is proposed, which uses random forest [27], extreme random regression tree [28] and GBDT regression algorithm [29] as the basic learning machines for the mixed gas data, and then merges each base learner algorithm according to the stacking model. Finally, the automatic grid search algorithm is applied to the stacking model for parameter tuning [30], and a concentration prediction algorithm for stacking model based on grid search is proposed.

The rest of this paper is organized as follows. Section 2 introduces the model construction of a mixture gas concentration prediction algorithm. Section 3 presents an automatic grid search method based on stacking for parameter tuning. Section 4 reports the experimental data and analyses the results of the proposed method. Finally, Section 5 concludes the paper.

## 2. Construction of Mixed Gas Concentration Prediction Model Based on Stacking

For the concentration regression prediction of mixed gas, model fusion based on a stacking algorithm is proposed. The base learning devices used are random forest, extreme random regression tree and GBDT regression algorithm.

### 2.1. Random Forest Principle

In machine learning, random forest is a classifier that contains multiple decision trees. Each decision tree is a classifier, and for an input sample, N trees will have N classification results. Random forest integrates all classification voting results and assigns the category with the most votes as the final output [31,32].

The random forest algorithm is composed of multiple decision trees, therefore, studying the random forest algorithm should understand the basic knowledge of decision trees first. The decision tree algorithm is a classic machine learning algorithm. The data structure of the decision tree is a tree structure. The tree structure has leaf nodes, non-leaf nodes and branches connecting child nodes. These three correspond to the classification results, intermediate decision nodes and classification features, respectively.

The specific implementation of the decision tree algorithm is as follows.

Set the training data set to $D=\{\left(x_{1},y_{1}\right),\left(x_{2},y_{2}\right),\ldots ,\left(x_{n},y_{n}\right)\}$, where $x_{i}={\left(x_{i,1},x_{i,2},...,x_{i,p}\right)}^{T}$. *p* is the total number of features, $y_{i}\in \left\{1,2,\ldots ,K\right\}$ is the class tag and i=1,2,…,N, where *N* is the size of the training sample. Based on all training samples $\left(x_{1},y_{1}\right),\left(x_{2},y_{2}\right),\ldots ,\left(x_{n},y_{n}\right)$, the algorithm repeats the following operations recursively for each node to be split until the termination condition is reached:

Use specific selection metrics to select the best attribute.

The optimal attributes are used to split the node to be split, so that the node to be split is divided into multiple child nodes, and corresponding to multiple regions. For example, binary division divides the node to be split into left and right child nodes.

When predicting *x*, perform a tree traversal operation on *x*, and enter a leaf node along the path from the root of the tree. Let *m* denote the leaf node where *x* falls, and the variable $y_{m_{1}},y_{m_{2}},\ldots ,y_{m_{n}}$ represent the corresponding value of training data in leaf node *k*.

For the regression problem, we need to predict the value of *x*, use Equation (1) for calculation, and calculate the average of the corresponding data for the regression prediction value of *x*.

For the classification problem, a voting algorithm is used to select the category with the highest number of votes in *m* as the category of *x*. If $y_{m_{i}}=y$, then $I(y_{m_{i}}=y)=1$; otherwise, $I(y_{m_{i}}=y)=0$.

(1) $$\begin{array}{c} \hat{h}\left(x\right)={\bar{y}}_{m}=\frac{1}{n}\sum_{i=1}^{n}y_{m_{i}} \end{array}$$

(2) $$\begin{array}{c} \hat{h}\left(x\right)=argmax_{y}\sum_{i=1}^{n}I\left(y_{m_{i}}=y\right) \end{array}$$

### 2.2. Extreme Random Regression Tree

**Figure 1:**
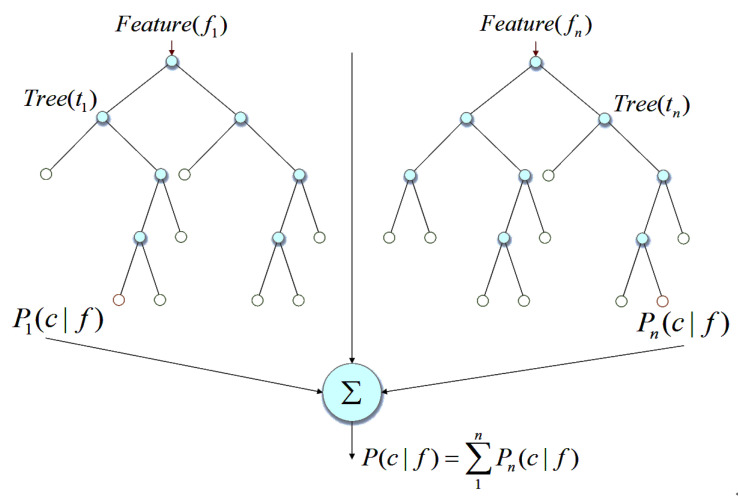
Schematic diagram of extreme random tree algorithm.

Extreme random tree (referred to as ET, also known as extreme random forest) is integrated by multiple decision trees which is an improvement of random forest. The generalization ability of extreme random trees is higher than that of random forests. In the extreme random tree algorithm, every decision tree uses all of the raw data, and the split node is selected randomly when the node is split, which enhances the randomness of the base classifier node split. While the random forest algorithm uses bootstrap sampling to generate training samples [33,34]. Extreme random tree has many excellent performances. For example, it is effective for processing a multi-dimension data set and does not require careful feature selection by the user; that is, the algorithm has the ability to automatically select features. Figure 1 is a schematic diagram of the extreme random tree algorithm.

The extreme random tree algorithm is represented by {E(K,X,D)}, where *E* represents the classifier model, *D* represents the original data sample and *K* represents the number of decision trees. For the samples to be classified, each decision tree in the extreme random tree outputs a prediction result. The extreme random tree uses voting to synthesize many results, and ultimately outputs the category of the sample.

The operation flow of the extreme random tree algorithm is given below:

(1) For each decision tree model in an extreme random tree, we use all training data for independent training.

(2) Generate a decision tree according to the CART algorithm [35]. Select *m* features from *M* features randomly in each splitting node during node splitting. At the same time, it is necessary to select appropriate attributes for node splitting referring to the feature selection metrics. Repeat this process until the decision tree growth stops.

(3) Repeat steps (1) and (2) for *K* iterations, and finally generate an extreme random tree model composed of *K* decision trees.

(4) Test the trained extreme random tree model through the test data, and finally generate the final classification result by voting.

### 2.3. Gradient Boosting Decision Tree Regression Algorithm

Each decision tree in the GBDT algorithm does not produce independent prediction results for observations. Each decision tree only learns a portion of the observation data, and the output of each decision tree needs to be accumulated as the final prediction of the observed value [36,37]. The GBDT regression algorithm includes three aspects: regression tree algorithm, gradient lifting and shrinkage.

#### 2.3.1. Regression Tree

The regression tree belongs to the field of decision tree and it is used for regression prediction. The classification decision tree is used for the classification problem. The decision tree uses a selection metric such as the Gini coefficient to split the node when the node is split. In addition, when a category of certain data is predicted, find the category with the highest number of votes where the data falls, and this category is used as the category of the test data. In contrast, in the regression problem, the problem of node splitting and regression prediction is also involved.

Since the regression problem is a continuous numerical prediction problem, for each node to be split, a method of minimizing the mean square error is used to find the split attribute. The regression tree algorithm needs to traverse each attribute, set various thresholds and calculate the value of the mean square error for each attribute, and then select the attribute with the smallest average error as the split attribute of the node.

When predicting the data, the data are traversed from the root of the tree. The average value of all data of the leaf node where the data fall is calculated as the predicted value.

#### 2.3.2. Gradient Lifting

The GBDT algorithm requires several iterations during training. In each iteration, a decision tree is trained, and the final prediction result is the sum of the predicted values of all decision trees. Specifically, in each iteration, the sum of the outputs of all previously trained decision trees is calculated. The residual between the cumulative sum and the real value is then obtained as the learning objective of the current decision tree.

Setting $T(x;\theta_{m})$ as the *m*th regression tree in the GBDT algorithm, the corresponding parameter is $\theta_{m}$, and there are *M* regression trees in the GBDT algorithm. The prediction function $f_{M}\left(x\right)$ is then the cumulative sum of the predicted values of *M* regression trees, which is:

(3) $$\begin{array}{c} f_{M}\left(x\right)=\sum_{m=1}^{M}T\left(x;\theta_{m}\right) \end{array}$$

Using boosting’s forward step-by-step algorithm, each round trains a new regression tree based on the results of the previous steps. At the *m*th iteration, there is:

(4) $$\begin{array}{c} f_{m}\left(x\right)=f_{m-1}\left(x\right)+T\left(x;\theta_{m}\right) \end{array}$$

After the end of the *m*th round, the prediction function is the cumulative sum of the predictions of the preceding *m* regression trees. By comparison with the true value *y*, the loss function can be obtained:

(5) $$\begin{array}{c} L\left(f_{m}\left(x\right),y\right)=L\left(f_{m-1}\left(x\right)+T\left(x;\theta_{m}\right),y\right) \end{array}$$

The value of the loss function is the parameter. In each iteration, the goal is to build a regression tree $T(x;\theta_{m})$ to minimize $L(f_{m}\left(x\right),y)$.

An optimization algorithm is utilized in order to minimize $L(f_{m}\left(x\right),y)$. The following uses the gradient descent method as an example to demonstrate the use of gradient boosting. The gradient descent method is a common and concise solution method which is briefly introduced below.

The goal of the gradient lifting algorithm is to minimize the objective function value f(ω) by changing the parameter ω, which is:

(6) $$\begin{array}{c} minf(\omega ) \end{array}$$

First, initialize the algorithm, randomly select the initial parameter $\omega_{0}$ and then perform multiple iterations. In each iteration, first calculate the gradient (corresponding to the downward direction of the function curve) $d_{i}=-\frac{\partial }{\partial_{\omega }}f\left(\omega \right)|\omega_{i}$, and then update the parameter ω according to the set step size ρ, that is: $\omega_{i+1}=\omega_{i}+\rho *d_{i}$. After *M* iterations, the algorithm returns the optimal solution $\omega^{*}$; letting $\omega_{0}=d_{0}$, $\omega^{*}$ is:

(7) $$\begin{array}{c} \omega^{*}=\sum_{i=0}^{M}\rho_{i}*d_{i} \end{array}$$

Using the lifting tree and the gradient descent method, the GBDT algorithm based on the gradient descent optimization algorithm can be produced. The process is as follows:

(8) $$\begin{array}{c} F_{k_{0}}\left(x\right)=0,k=1,2,\ldots ,K \end{array}$$

Traverse m=1,2,…,M, then:

(9) $$\begin{array}{c} p_{k}\left(x\right)=\frac{exp(F_{k}\left(x\right))}{\sum_{l=1}^{K}exp\left(F_{l}\left(x\right)\right)},k=1,2,\ldots ,K \end{array}$$

Traverse k=1,2,…,K, then:

(10) $$\begin{array}{c} \vec{y_{ik}}=y_{ik}-p_{k}\left(x_{i}\right),i=1,2,\ldots ,N \end{array}$$

(11) $$\begin{array}{c} {\left\{R_{klm}\right\}}_{l=1}^{L}=L-{\left\{\vec{y_{ik}},x_{i}\right\}}_{1}^{N} \end{array}$$

(12) $$\begin{array}{c} \gamma_{klm}=\frac{K-1}{K}*\frac{\sum_{x_{i}\in R_{klm}}\vec{y_{ik}}}{\sum_{x_{i}\in R_{klm}}|\vec{y_{ik}}\left|(1-\right|\vec{y_{ik}}\left|\right)} \end{array}$$

(13) $$\begin{array}{c} F_{km}\left(x\right)=F_{k,m-1}\left(x\right)+\gamma_{klm}^{l(x\in R_{klm})} \end{array}$$

#### 2.3.3. Shrinkage

The idea of shrinkage is reflected in the fact that the algorithm takes a small step at a time and gradually approximates the result, rather than approaching the real result at the beginning, which is beneficial to avoid overfitting. In the GBDT regression algorithm, it is not necessary for the first regression tree to offer a very good prediction effect, but through continuous iteration, the prediction results of multiple decision trees are accumulated step by step to approximate the true value.

### 2.4. Stacking Model Construction

The main idea of the stacking algorithm is to combine multiple weak learning devices into a strong learning machine, and then add another learning device layer on the basis of the strong learning machine. This model is composed of a layer of learners in addition to another layer of learners, which can achieve more accurate prediction of the data set. Generally, we call the weak learning device the primary learner, and the strong learning machine composed of the weak learning devices at the first layer is called the secondary learner; both learners are integrated into a new learner through certain rules to form a more powerful final learner to predict and analyze results.

**Figure 2:**
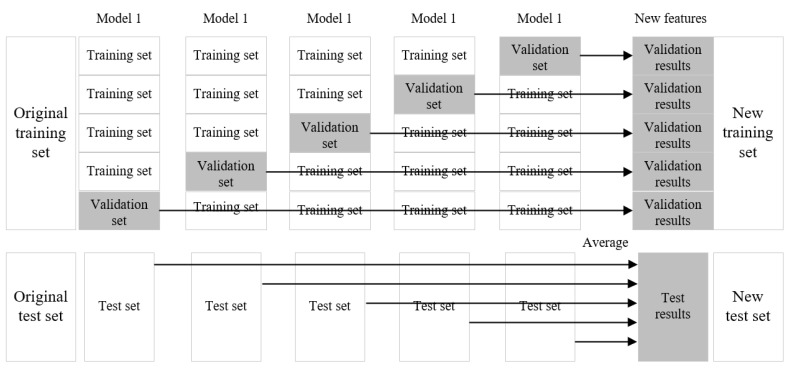
Stacking algorithm implementation process.

In the case of practical application, different basic learning devices can be used to form the stacking model. The implementation process of the stacking model is shown in Figure 2.

The entire implementation process involves taking the verification results of all basic learner models that have passed the training as the new training set, and the results of the test set as the new test set. This is the case for one cycle, the stacking model lets all data sets be learned by the base learning devices, and uses cross-validation to form a new training set, and uses the average method to form a new test set. The new training set are the verification results obtained from the original training set. The new test set is formed by averaging the prediction results of the original test set.

The stacking model uses a layered structure. We only analyze the second-level stacking. Suppose that we have three base models, $M_{1}$, $M_{2}$ and $M_{3}$. For the basic model $M_{1}$, the training set is trained, and then used to predict and test the label, and the results of the predicted training set and test set are used as $P_{1}$ and $T_{1}$.

(14) $$\left(\begin{array}{c} \vdots \\ P_{1}\\ \vdots \\ \vdots \end{array}\right)\left(\begin{array}{c} \vdots \\ T_{1}\\ \vdots \\ \vdots \end{array}\right)$$

For the base models $M_{2}$ and $M_{3}$, such steps are also performed to obtain $P_{2}$, $T_{2}$, $P_{3}$ and $T_{3}$. We combine $P_{1}$, $P_{2}$, $P_{3}$ and $T_{1}$, $T_{2}$, $T_{3}$ to produce a new training set train2 and test set test2.

(15) $$\left(\begin{array}{c} \vdots \\ T_{1}\\ \vdots \\ \vdots \end{array}\right)\left(\begin{array}{c} \vdots \\ T_{2}\\ \vdots \\ \vdots \end{array}\right)\left(\begin{array}{c} \vdots \\ T_{3}\\ \vdots \\ \vdots \end{array}\right)\Rightarrow \overset{New\;test\;set}{\overset{︷}{\left(\begin{array}{ccc} \vdots & \vdots & \vdots \\ T_{1} & T_{2} & T_{3}\\ \vdots & \vdots & \vdots \\ \vdots & \vdots & \vdots \end{array}\right)}}$$

**Figure 3:**
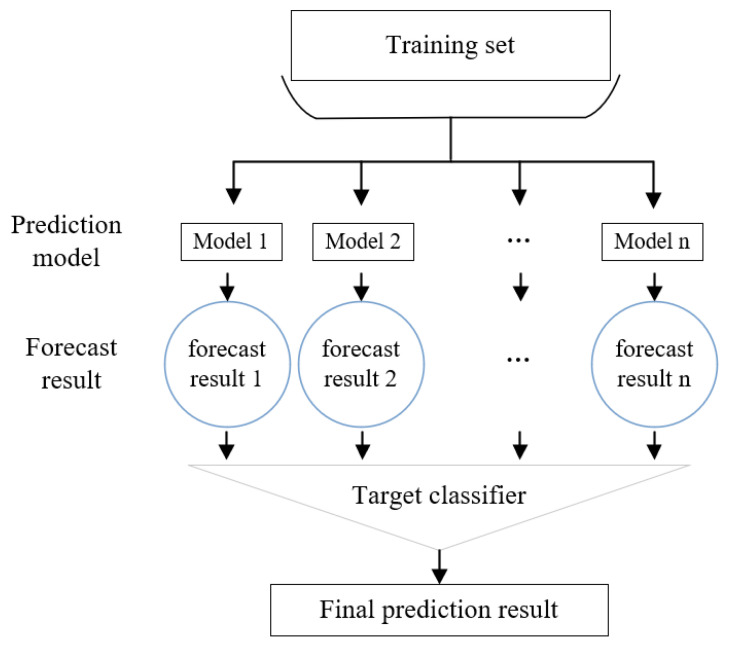
Stacking algorithm implementation form.

We then use the second layer learner $M_{4}$ to train train2 and predict test2 to obtain the final regression prediction result. One notable aspect is that the features in train2 and test2 are the results predicted by y in the first layer. The implementation flowchart of the stacking algorithm is shown in Figure 3.

**Figure 4:**
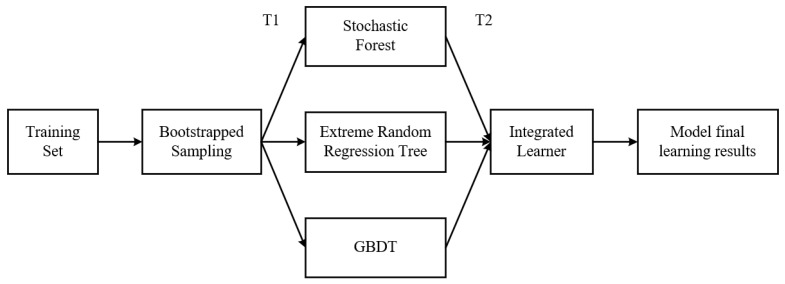
Model construction of stacking algorithm.

The stacking model combines different models to realize the fusion of models. First, a new model is trained to obtain a final output by taking the output results of different models trained in the previous round as a new input. In this paper, the random forest algorithm, the GBDT regression algorithm and the extreme random tree algorithm are used as the base regression algorithms for combining the algorithms. In theory, stacking can represent the integration method of the three basic algorithms mentioned above. The following Figure 4 is the construction of the stacking algorithm involved in this paper. First, the entire data set is classified into training set and test set. Each training set is obtained by bootstrapped sampling on the whole training data set, and a series of basic learning models are obtained, which are called T1 learners. The output of T1 learner is then used to train T2 learner. In T1 learner, the method of 10-fold cross-validation is used to train the learner. Each learner in T1 is trained according to the remaining 9 pieces and it is tested on the test set. The output of these learners is then used as input to train T2 learners on the entire training set.

## 3. Automatic Grid Search Parameter Tuning of Stacking

The essence of the grid search method is to divide all of the parameters that need to be searched into a grid with the same length according to the established spatial search range and the proposed coordinate system. Each point in the coordinate system represents a set of parameters that must be verified, and its performance can be verified and analyzed by bringing all of the points in this given interval into the tuning system. We refer to the best performance of the entire system as the optimal parameter [38]. The main parameters to be adjusted of random forest, extreme random tree and GBDT regression algorithm which are selected as the base learning devices are tested below.

The data used for parameter tuning is the feature data vector composed of the gas concentration and the feature values of each sensor response in the sensor array. Please refer to Table 4 of “Experimental data” in Section 4. First, the random forest algorithm applies the bagging model. The main sensitivity parameters to be adjusted by their own framework are the number of decision trees “n_estimators”, the maximum characteristic number of decision trees “max_features”, the minimum number of samples needed for node partition “min_samples_split”, the number of minimum sample leaf nodes “min_samples_leaf”, the maximum number of leaf nodes “max_leaf_nodes” and the maximum depth of decision tree “max_depth”, as shown in Table 1. If these parameters are not optimized, there will be personal subjective interference and the highest performance of the algorithm will not be achieved.

**Figure 5:**
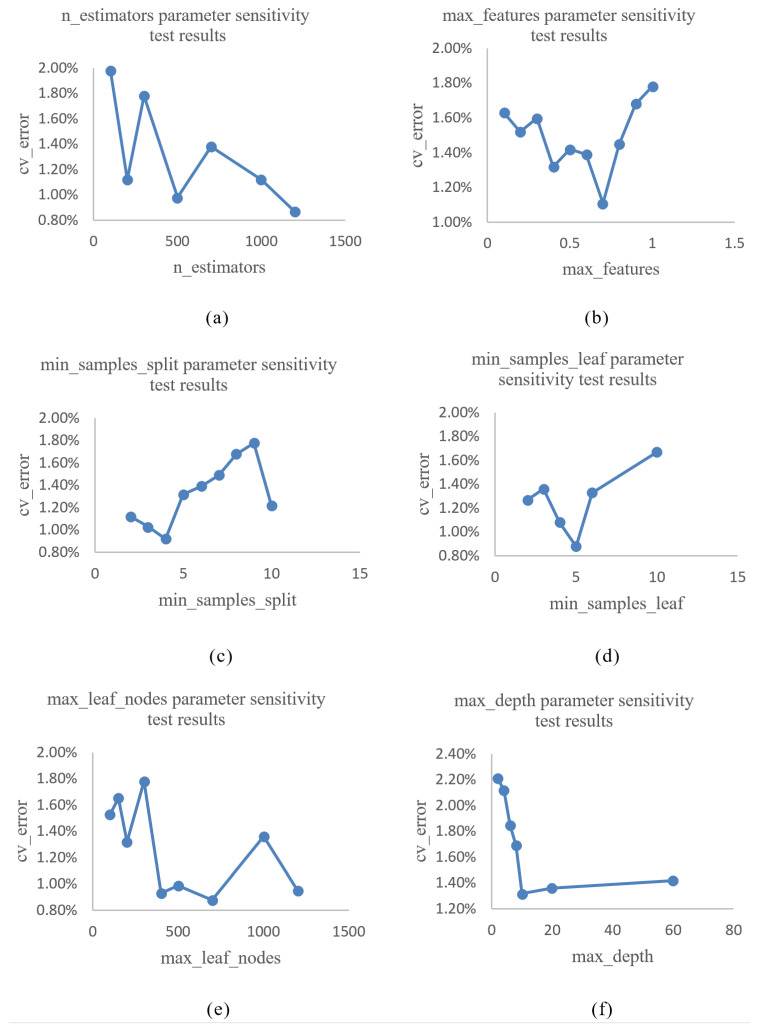
Random forest parameter sensitivity test results. (**a**) n_estimators parameter sensitivity test results. (**b**) max_features parameter sensitivity test results. (**c**) min_samples_split parameter sensitivity test results. (**d**) min_samples_leaf parameter sensitivity test results. (**e**) max_leaf_nodes parameter sensitivity test. (**f**) max_depth parameter sensitivity test.

Here, taking the mean of 10 times 10-fold cross-validation error rate as the evaluation standard, the smaller the value, the higher the accuracy of the algorithm. With the premise of ensuring that the other parameters are default parameters, we select different single parameter values to draw an image. The mean value of 10 times 10-fold cross-validation error rate (cv-error) is displayed in the vertical coordinate, and different values of parameters are displayed in the horizontal coordinate in this image. Observing the trend of the image, if the trend of error rate is obvious or follows a certain value, the error rate is essentially unchanged. The parameters are then considered to be insensitive. If the error rate is unstable with the change of parameters and the minimum value appears in many places, the parameters are considered to be sensitive. The sensitivity results of each parameter for the algorithm are shown in Figure 5.

As can be observed from Figure 5, the results of sensitivity testing show that when the parameter min_samples_split is 4, the error rate is the lowest. When the parameter min_samples_leaf is 5, the error rate is the lowest. When the parameter max_depth is 10, it no longer greatly influences the error rate. The experimental results show that these three parameters min_samples_split, min_samples_leaf and max_depth are insensitive and do not need to tune. The values can be min_samples_split = 4, min_samples_leaf = 5 and max_depth = 10.

It can be observed from Figure 5 that the influence of n_estimators, max_features and max_leaf_nodes on the error rate is uncertain, and it is determined that these three parameters are sensitive to the algorithm. It is necessary to perform parameter tuning. The parameters are optimized by automatic grid search algorithm, and the final results are as follows: n_estimators = 700, max_features = 0.4 and max_leaf_nodes = 300. After the parameter optimization of the automatic grid search, the 10-fold cross-validation error rate of the random forest algorithm changed from 3.56% to 1.45%, a reduction of 2.11%.

According to the classical top-down approach, the extreme random tree algorithm model constructs a series of sets of extreme random decision tree models. Each tree obtains the bifurcation value completely randomly, to realize the bifurcation of the decision tree. The extreme random tree algorithm is an integration of multiple decision trees, which is similar to the random forest algorithm. The difference is the source of model training data; the former uses all of the data, while the latter is sampled by bootstrap method. The main sensitivity parameters of the extreme random tree algorithm model are the same as those of the random forest, as shown in Table 1. Moreover, after 10 iterations of parameter sensitivity tests of 10-fold cross-validation, the sensitive parameters of the extreme random tree algorithm are the same as those of the random forest algorithm. By the same token, we can obtain that the tuning parameters are n_estimators = 300, max_features = 0.3 and max_leaf_nodes = 500. After the parameter optimization of the automatic grid search, the 10-fold cross-validation error rate was changed from 3.07% to 1.28%, a reduction of 1.79%.

The following are the main parameters in the GBDT regression algorithm. As a result that the GBDT regression algorithm adopts the Boosting model, its main parameters are divided into two parts: the framework parameters of the GBDT regression algorithm and the parameters of the decision tree itself. The frame parameters of GBDT mainly include sampling proportion “subsample” and learning weight “learning_rate”. The GBDT regression algorithm uses a sample that is not replaced, and when the sampling proportion is 1, it means that all of the samples are sampled, which is equivalent to the non-sampling. Thus, the parameter “subsample” is generally less than 1, and typically between 0.5 and 0.8. The parameter “earning_rate” represents the reduced-weight parameter of the learner, also known as the step size. The parameters of the decision tree are similar to those of the random forest, and the detailed parameters are shown in Table 2 below.

The basic learner of GBDT is also a decision tree; thus, its parameters related to decision trees are no longer subject to sensitivity testing. Referring to the random forest algorithm, continuously select n_estimators, max_features and max_leaf_nodes as sensitive parameters to participate in the optimization of the automatic grid search algorithm. In this paper, only two parameters of the framework parameter in the GBDT regression algorithm are discussed, they are “subsample” and “learning_rate”. The two parameters are directly used as the tuning parameters for the grid search. The parameters that need to be adjusted are n_estimators, max_feature, max_leaf_nodes, subsample and learning_rate, and the values after final determination are 300,0.3,500,0.8,0.5, respectively. After the parameter optimization of the automatic grid search, the 10-fold cross-validation error rate was changed from 3.12% to 1.63%, a reduction of 1.49%. It can be determined that these three algorithms after the parameter optimization exert a positive effect on the regression prediction of the mixed gas. The random forest algorithm, the extreme random tree algorithm and the GBDT regression algorithm, which have been optimized by the parameters, will be used as the base learning devices for the model fusion based on the stacking algorithm, and this model is used to predict the concentration of mixed gas in the final stacking model.

## 4. Prediction Results and Analysis of Mixed Gas Concentration

### 4.1. Evaluating Indicator

Two kinds of algorithm consideration index are selected to consider in the paper. In the first category, the mean squared error MSE, goodness of fit $R^{2}$, the median absolute error MedAE and the average absolute error MAE are used to evaluate the common evaluation indexes of the regression algorithm. The mean square error of MSE represents the expected value of the error square between the predicted value and the real value. The smaller the MSE, the better the accuracy of the algorithm. $R^{2}$ is the goodness of fit in regression prediction, which reflects the interpretability of independent variables to dependent variables. The value is less than or equal to 1, and the larger $R^{2}$ is, the better. If the value is less than 0, the prediction algorithm is not as good as the benchmark algorithm. If the sum of squares of residual errors is simply used, it will be affected by the absolute value of the dependent variable and the independent variable, which is not conducive to the relative comparison between different models. This problem can be solved by evaluating the goodness of fit. The median absolute error MedAE represents the intermediate value of each absolute error, which is very suitable for the data set of outliers. The average absolute error MAE represents the average value of the absolute error, which can better reflect the actual situation of the predicted value error. In the second category of consideration index, algorithm fitting situation is displayed in the form of a scatter plot. The true value of the scatter graph is the transverse coordinate, and the predicted value is the longitudinal coordinate. On the line y=x, the predicted value is the same as the real value, and the more points which gather on the y=x line, the better the fitting degree of the algorithm and the higher the prediction accuracy.

The four evaluation indicators are defined as follows:

(16) $$\begin{array}{c} MSE=\frac{1}{n}\sum_{i=1}^{n}{\left(y_{i}-\hat{y_{i}}\right)}^{2} \end{array}$$

(17) $$\begin{array}{c} R^{2}=1-\frac{\sum_{i=1}^{n}{\left(\hat{y_{i}}-{\bar{y}}_{i}\right)}^{2}}{\sum_{i=1}^{n}{\left(y_{i}-{\bar{y}}_{i}\right)}^{2}} \end{array}$$

(18) $$\begin{array}{c} MedAE\left(y,\hat{y}\right)=median(|y_{1}-\hat{y_{1}}\left|,\ldots ,\right|y_{n}-\hat{y_{n}}\left|\right) \end{array}$$

(19) $$\begin{array}{c} MAE\left(y,\hat{y}\right)=\frac{1}{n}\sum_{i=1}^{n}\left|y_{i}-\hat{y_{i}}\right| \end{array}$$

In the equations above, *n* denotes the number of data sets, $\hat{y_{i}}$ denotes the evaluation prediction results, ${\bar{y}}_{i}$ denotes the average value of the prediction results and $y_{i}$ is the true value.

### 4.2. Experimental Data

The aim of this paper was a classification and concentration detection algorithm for mixed gas. The data set used is obtained from the acquisition of measurements of a metal oxide semiconductor sensor (MOS) array composed of TGS sensors from FIGARO Company. The TGS sensor has high sensitivity to flammable and explosive gases. It is a thick film MOS sensor with short response time, low power consumption, low cost, small size and good long-term stability. A simple circuit can be used to have good sensitivity to the gas to be measured, which is very suitable for use in toxic and explosive leak detectors. By establishing a machine olfactory system of multiple sensor types, the multivariate response stimulated by methane, ethylene and carbon monoxide gas is obtained.

**Figure 6:**
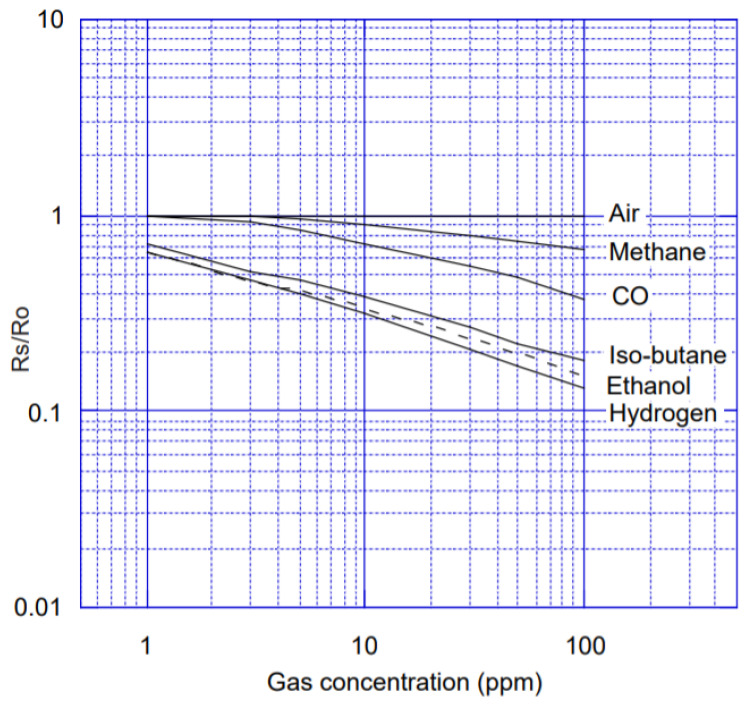
TGS2600 sensor characteristic diagram.

The experimental platform utilized a wind tunnel with two independent gas sources. Each source was controlled independently to release the selected volatiles, which generated different concentration levels in the sensors’ position. The wind generator created a turbulent flow that constantly displaced the introduced volatiles towards the exhaust outlet. The detection platform was composed of 8 MOX gas sensors that generate a time-dependent multivariate response to the different gas stimuli. The operating temperature of the sensors was controlled by the built-in heater, which was kept at a constant voltage of 5 V. Figure 6 is a picture in the technical manual of TGS2600, describing the change of resistance with gas concentration. $R_{S}$ is the sensor resistance value in various gas concentrations. $R_{0}$ is the resistance value of the sensor in the clean air.

The data set collected by the machine olfactory data acquisition system includes 30 categories [39], and each label consists of 6 experiments with different data sets. As shown in Table 3, “n” means the concentration is 0, “L” means low concentration, “M” means medium concentration, “H” means high concentration. The total of 180 data sets are mixtures of ethylene-carbon monoxide and ethylene-methane.

The mixed gas data set is collected by the sensor array composed of 8 sensors, and the sampling frequency is set to 50 Hz. The duration of the data sampling phase is 300 s. There is no ventilation for the first 60 s. From 60 s to 240 s, the mixed gas with set concentration ratio is passed into the gas chamber. From 240 s to 300 s, no mixed gas was introduced. The data sets are stored according to the time rule. Each data set contains 11 columns of data: time (s), temperature, humidity (%) and TGS2600, TGS2612, TGS2611, TGS2610, TGS2602, TGS2602, TGS2620, TGS2620 data. The data collected by the sensor are the voltage values ($V_{RL}$) of the external load resistance $R_{L}$ and the sensor resistance is $R_{S}$. The loop voltage is $V_{c}$ and the relationship between $V_{RL}$ and $R_{S}$ is:

(20) $$\begin{array}{c} V_{RL}=\frac{R_{L}}{R_{S}+R_{L}}\times V_{c} \end{array}$$

**Figure 7:**
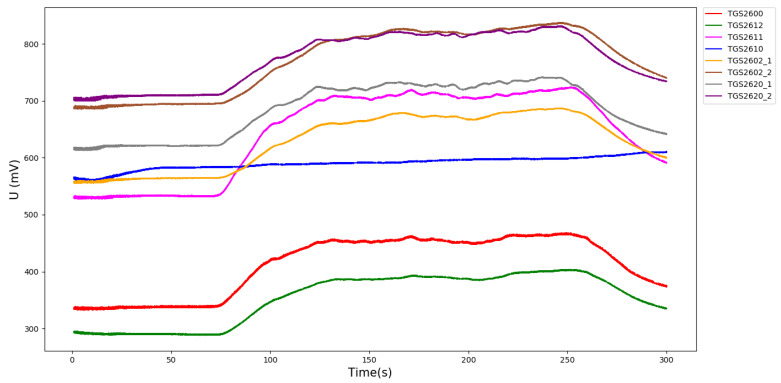
Sensor response graph under Et_L_Me_H tag.

The loop voltage $V_{c}$ is unchanged. After contacting the target gas, the resistance value changes, the resistance value of the sensor $R_{S}$ decreases, the resistance value of the load $R_{L}$ increases, thus, the voltage value $V_{RL}$ increases. Therefore, the data set of the voltage value $V_{RL}$ can be used to detect the type of mixed gas. The sensor response diagram of the experiment is shown in Figure 7 (in the case of Et_L_Me_H, that is under conditions of low concentration of ethylene and high concentration of methane, there are 26,887 columns in this data set), the abscissa is time and the ordinate is the converted sensor voltage value.

Different types of sensors respond differently to the same mixed gas. Even if it is the same type of sensor, there will be differences due to individual differences in the sensors and different positions. The above-mentioned sensors all respond to a variety of reducing gases, but the main detection objects for each sensor are different. Therefore, the use of sensor arrays is more universal. In the case of the data set for gas concentration detection, the purpose is to detect the concentrations of ethylene and methane. Table 4 lists the gas characteristics and concentration values in parts per million by volume (ppm). The resulting data set includes 13,910 time series collected within 36 months [40].

### 4.3. Analysis of Results

Use the mixed gas data set to perform regression prediction of gas concentration, and obtain the gas concentration prediction results of carbon monoxide and ethylene, respectively. Compare the results with blending algorithm [41], bagging algorithm [42], averaging algorithm and SVR algorithm [43].

#### 4.3.1. Blending Algorithm Principle

The blending process is very similar to the stacking process. The main difference lies in the different training sets. The stacking training set generation process is: use the K-fold cross-validation method to obtain predicted values, and then generate the features as training set needed in the second stage based on these predicted values. In contrast, the training set of blending is derived from the holdout set. In short, blending uses disjoint data sets for the training process of different model layers.

For the first-level model, the blending algorithm needs to divide the data into a training set (train_set) and a validation set (val_set), and select multiple homogeneous or heterogeneous models as the basic models. Then use the training set to train these models, and verify the trained model on the validation set to obtain the predicted features which will serve as the training set for the second layer.

The second-layer of the blending model is a common computational learning method, and its training set is the predicted features obtained from the first layer. There are two layers in the testing process of the blending model. In the first layer, the trained model is used to predict the test data to obtain the predicted characteristics of the test set; in the second layer, the trained single-layer perceptron is used to predict the predicted characteristics to obtain the final prediction result.

#### 4.3.2. Bagging Algorithm Principle

The Bootstrap method is a typical sampling method, thus, the training set is replaced and repeatedly sampled. The bagging algorithm is an integrated learning algorithm using the Bootstrap method. Through Bootstrap sampling on the training set, multiple independent machine learning models can be obtained. Random forest algorithm is a classic algorithm that applies bagging theory for integrated learning.

Taking the random forest algorithm as an example, the following shows how to use bagging algorithm for integrated learning. Through Bootstrap sampling, independent data are allocated to each decision tree for training, so that each decision tree is independent. When a new sample is predicted, the new sample is input into each decision tree model, and their results are integrated to give the prediction result of the new sample. The specific algorithm flow is as follows:

(1) Decision tree training data division stage: Use the Bootstrap method on the original data set to obtain multiple independent training data for training each decision tree.

(2) Decision tree training stage: Each decision tree is trained independently, and the information gain is used as an attribute selection metric to split the node until all the data in the node belong to the same category, or the depth limit is reached to stop the split, the whole process does not perform the pruning operation.

(3) Model summary stage: Summarize the results of all decision trees. For classification problems, the voting method is used, and the category with the highest number of votes is used as the predicted category; for regression problems, the average value of the output results of each decision tree is used as the final predicted value.

#### 4.3.3. Averaging Algorithm Principle

Averaging also combines multiple different learners, but the combination rules are different from the previous algorithms. The main idea is to average the results between different algorithms. For regression prediction, models composed of different algorithms are used as base learners, and the prediction results of each base learner are averaged as the prediction results of the overall model.

According to the evaluation index parameters, the specific results of each algorithm are shown in Table 5 and Table 6. Table 5 shows the regression prediction results for carbon monoxide gas. Table 6 presents the regression prediction results for ethylene gas.

Through Table 5, it can be determined that for the prediction of carbon monoxide, the highest fit value of the stacking algorithm is 0.9901, which indicates that the fitting condition of the algorithm is very good. The fit value of the averaging algorithm is 0.9887, the fit value of the blending algorithm is 0.9873 and the fit value of the bagging algorithm is 0.9831, which is the lowest among the above algorithms except for SVR. The median absolute error of stacking is 1.8442, which is 2.6136 lower than that of the bagging algorithm, 2.1133 lower than that of the averaging algorithm, 0.1257 lower than that of the blending algorithm. The average absolute error of stacking is 4.1162, which is 2.4229 lower than that of the bagging algorithm, 1.6466 lower than that of the averaging algorithm, 0.7354 lower than that of the blending algorithm. The goodness of fit, median absolute error, average absolute error and mean square error for the stacking algorithm are much higher than those of the SVR algorithm.

Through the comparison between Table 5 and Table 6, it can be determined that the prediction results of ethylene concentration are not different from those of carbon monoxide concentration prediction. The prediction results of the stacking algorithm are the best for the prediction of ethylene. The goodness of fit of the stacking algorithm is 0.9913, the median absolute error is 1.7842 and the average absolute error is 4.0459. The goodness of fit of the bagging algorithm is 0.9820, the median absolute error is 4.8566 and the average absolute error is 6.6022. The goodness of fit of the averaging algorithm is 0.9846, the median absolute error is 4.1125 and the average absolute error is 5.9546. The goodness of fit of the blending algorithm is 0.9900, the median absolute error is 1.8457 and the average absolute error is 4.6251. Among them, the averaging algorithm is better than the bagging algorithm overall, but worse than blending as a whole. The prediction effect of SVR is much worse than that of the stacking algorithm.

**Figure 8:**
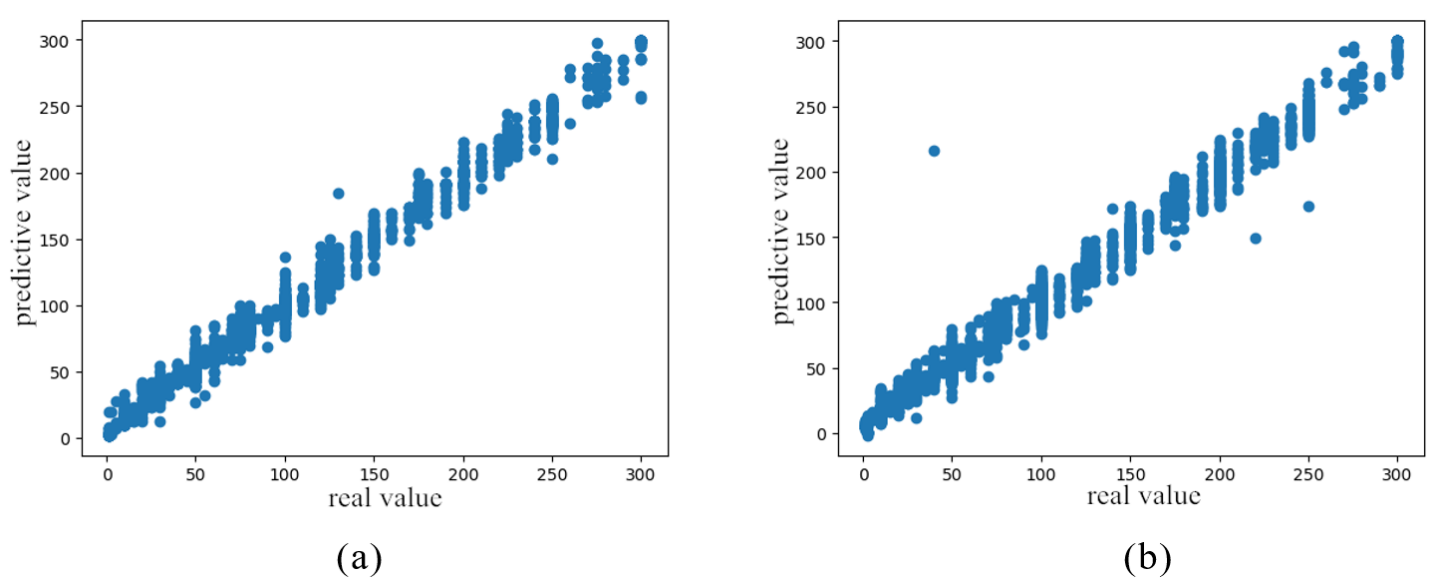
Comparison diagram of fitting algorithm. (**a**) Prediction result before stacking parameter optimization. (**b**) Prediction result after stacking parameter optimization.

In order to further analyze the prediction of mixed gas concentration, the prediction results are analyzed more clearly by visual presentation. Figure 8 shows the comparison of the effects of carbon monoxide concentration prediction parameters before and after tuning.

**Figure 9:**
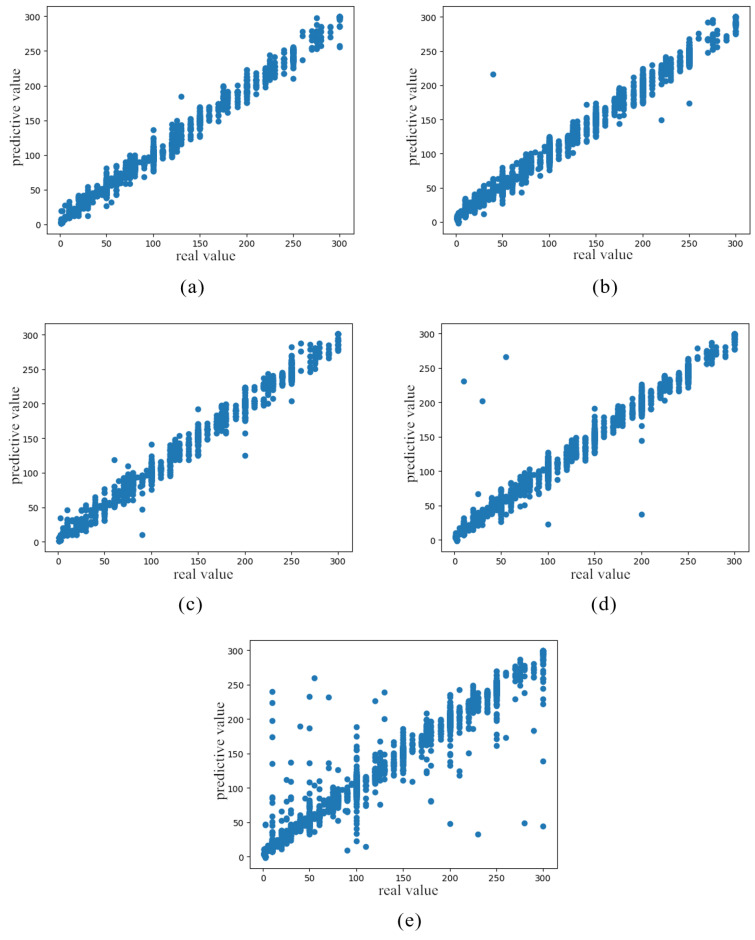
Comparison diagram of fitting algorithms. (**a**) Stacking algorithm prediction result. (**b**) Blending algorithm prediction result. (**c**) Bagging algorithm prediction result. (**d**) Averaging algorithm prediction result. (**e**) SVR algorithm prediction result.

At the same time, the prediction of carbon monoxide is shown in Figure 9, in which the transverse coordinates are the actual test values, the longitudinal coordinates represent the values of the predicted results, and the four regression algorithms are compared to characterize the prediction effect.

From the analysis of Figure 9, it can be determined that the graphics are close to the line of y=x, which is consistent with our expectation. It can be clearly observed that the fitting effect of the stacking algorithm is the best with respect to predicting the gas concentration value. The fitting effect is more concentrated on a straight line, while the dispersion rate of the predicted value of the SVR algorithm is the largest, which also explains the reason for the larger mean square error.

## 5. Conclusions

In this paper, the gas concentration prediction algorithm based on the stacking model was proposed, and the mixed gas data were modeled and analyzed with random forest, extreme random tree and GBDT regression algorithms. Parameter tuning based on grid search was conducted for the base learner used in the stacking model, and the optimal parameter was selected by 10-fold cross-validation error rate. Finally, the overall error rate was reduced by automatic grid searching of the tuned stacking model. The simulation methods were selected to verify and calculate the regression prediction indexes of the four models, including the median absolute error, average absolute error, mean square error and goodness of fit. The stacking model exhibited the best prediction effect for carbon monoxide and ethylene, with goodness of fit values of up to 0.9901 and 0.9913, respectively. In addition, it is verified by fitting the curve. Both of the above results indicate that the proposed stacking model based on stacking exhibits a better fitting effect and is more suitable for gas concentration prediction than other comparison algorithms. The proposed algorithm exerts an obvious effect on the precision of regression fitting and is suitable for concentration prediction, which provides a reference for the machine olfactory algorithm.

## Figures and Tables

**Table 1:** Common parameter settings for random forests.

Random Forest Parameters	Parameter Value
n_estimators	100,200,300,500,700,1000,1200
max_features	0.1,0.2,0.3,0.4,0.5,0.6,0.7,0.8,0.9
min_samples_split	2,3,4,5,6,7,8,9,10
min_samples_leaf	2,3,4,5,6,10
max_leaf_nodes	100,150,200,300,400,500,700,1000,1200
max_depth	2,4,6,8,10,20,60

**Table 2:** Common parameter settings for gradient boosting decision tree (GBDT).

GBDT Parameters	Parameter Value
subsample	0.5,0.6,0.7,0.8
learning_rate	0.1,0.2,0.3,0.4,0.5,0.6,0.7,0.8,0.9,1.0
n_estimators	100,200,300,500,700,1000,1200
max_features	0.1,0.2,0.3,0.4,0.5,0.6,0.7,0.8,0.9
min_samples_split	2,3,4,5,6,7,8,9,10
min_samples_leaf	2,3,4,5,6,10
max_leaf_nodes	100,150,200,300,400,500,700,1000,1200
max_depth	2,4,6,8,10,20,60

**Table 3:** Data set category information table.

Gas	Ethylene	n	L	M	H
	n	−	6	6	6
Carbon monoxide	L	6	6	6	6
	M	6	6	6	6
	H	6	6	6	6
	n	−	6	6	6
Methane	L	6	6	6	6
	M	6	6	6	6
	H	6	6	6	6

**Table 4:** Data set concentration information table.

Gas	Concentration (ppm)
Ethylene	5,10,15,20,25,30,35,40,50,60,70,75,80,90,100,110,120, 125,130,140,150,160,170,175,180,190,200,210,220,225,230, 240,250,260,275,285,300
Methane	5,10,13,20,25,30,35,40,45,50,60,70,75,80,90,100,110, 120,130,140,150,160,175,180,190,200,210,220,225,230,240, 250,275,280,295,300

**Table 5:** Carbon monoxide prediction results.

Carbon	Stacking	Blending	Bagging	Averaging	SVR
MedAE	1.8442	1.9699	4.4578	3.9575	4.2109
MAE	4.1162	4.8516	6.5391	5.7628	8.7930
MSE	66.5157	85.7748	113.2176	75.7827	404.2604
$R^{2}$	0.9901	0.9873	0.9831	0.9887	0.9411

**Table 6:** Ethylene prediction results.

Ethylene	Stacking	Blending	Bagging	Averaging	SVR
MedAE	1.7842	1.8457	4.8566	4.1125	4.6771
MAE	4.0459	4.6251	6.6022	5.9546	9.2911
MSE	65.2454	83.0125	114.2146	75.8315	543.3516
$R^{2}$	0.9913	0.9900	0.9820	0.9846	0.8945

## Data Availability

Not applicable.
